# Evidence for a role of adverse sarcomere signaling in atrial fibrillation induction

**DOI:** 10.1016/j.jmccpl.2026.100866

**Published:** 2026-09-03

**Authors:** Mark D. McCauley, R. John Solaro

**Affiliations:** a Department of Medicine, Section of Cardiology, Center for Cardiovascular Research, University of Illinois Chicago, Chicago, IL, United States of America; b Jesse Brown VA Medical Center, Chicago, IL, 60612, United States of America; c Department of Physiology and Biophysics and Center for Cardiovascular Research, University of Illinois Chicago, Chicago, IL, 60612, United States of America

**Keywords:** Atrial fibrillation, Atrial cardiomyopathy, Sarcomere, Titin, Myosin

## Abstract

Innovative therapies are needed in the face of a lack of a comprehensive, in-depth understanding of the complex mechanisms underlying atrial fibrillation (AF). There is recognition of a need for deeper understanding and novel therapeutic approaches, especially in atrial cardiomyopathy (ACM). ACM is considered a separate entity characterized by structural, architectural, contractile, or electrophysiological changes, increasing the potential for clinical atrial pathology. Evidence indicating a role for ACM in atrial disorders is that, in some cases, thromboembolic risk and persistent AF continue despite treatments such as ablation of isolated regions of the pulmonary vein known to set off AF. Here we review a possible role for modifications at the level of atrial sarcomeres as a common mechanism inducing genetic and acquired AF/ACM. Our review indicates the likelihood that these modifications induce adverse signaling resulting in fibrosis, inflammation, neuro-humoral activity, and arrhythmias, in turn promoting atrial pathology. We discuss genetic and acquired ACM through the lens of a new era of understanding of sarcomere control mechanisms. This advanced understanding has not been thoroughly discussed previously in the case of atrial sarcomeres. Significant new evidence has revealed atrial sarcomere specific mechanisms with the potential for novel control of the pressure-volume relation that is vulnerable in ACM. These mechanisms in atrial sarcomeres have taken on new significance with the identification and clinical development of small-molecule sarcomere activators and inhibitors with possible use and further development in ACM therapy.

## Highlights

• Atrial cardiomyopathy (ACM) is a key contributor to persistent atrial fibrillation (AF).
• Sarcomere protein expression profiles contribute to atrial electrical stability and pressure-volume relationships.
• Mechanisms controlling atrial sarcomeres are vulnerable in AF/ACM.
• Understanding atrial sarcomere function may help with future potential therapies in ACM.

## 1 Introduction

Research on acute atrial fibrillation (AF) and thromboembolic risk has traditionally focused on membrane-dependent ionic mechanisms controlling tension and conduction [1], [2], [3], [4], [5]. Pulmonary vein isolation effectively reduces AF triggers, yet many patients continue to experience persistent AF and thromboembolic risk, highlighting the need for new therapeutic strategies [1], [5]. Increasing evidence implicates atrial cardiomyopathy (ACM) as a key contributor to persistent AF [6], [7], [8], [9], [10], [11]. Studies indicate that ACM may be causal in cardioembolic stroke independently of AF [12]. ACM is defined as structural, architectural, contractile, or electrophysiological atrial alterations with clinical consequences, although its underlying mechanisms remain poorly understood [9].

While atrial hypo-contractility has long been associated with AF and thromboembolism, the precise mechanisms driving this dysfunction are unclear [7], [13]. Here we focus on emerging evidence indicating a potential role of homeostatic mechano-signaling. We review evidence that disrupted mechanical signaling may promote adverse mechano-electrical signaling and remodeling in both genetic and acquired ACM signaling [14], [15], [16], [17]. A central theme in our review is the consideration of mechano-signaling associated with the strain, stretch, and shear signals arising from the state of sarcomere Z-discs, M-bands, and titin. These structures can transmit mechanical signals through cytoskeletal and nuclear connections, altering electrical stability, mechanics, and gene expression [14], [18]. In turn, mechanical signals arising from changes in preload and afterload are transmitted to the sarcomeres via integrins, membranes, and intercalated disks, and ultimately to the nucleus, promoting fibrosis and cellular remodeling.

We survey studies demonstrating that dysregulated signaling to and from atrial sarcomeres represents a shared pathogenic pathway increasing atrial structural remodeling and electrophysiological instabilities in genetic and acquired ACM. These studies indicate that sarcomere functional flexibility in tension and dynamics is critical for adapting to variable atrial stresses, and loss of this flexibility contributes to acquired and genetic ACM [19], [20], [21]. Insights from familial hypertrophic and dilated cardiomyopathies support this model, as mutations produce persistent contractile and electrical imbalance, mechano-energetic uncoupling, altered Ca^2+^ sensitivity, and impaired regulatory mechanisms such as membrane stability and tension affecting stretch-activated channels, length-dependent activation and phosphorylation [19], [22], [23], [24]. Despite these advances in understanding, atrial sarcomere biology and mechanics remain underexplored compared with ventricular biology and mechanics. This review outlines distinct molecular features and function of atrial sarcomeres of potential significance in health and in pathogenic mechanisms in AF/ACM.

## 2 Distinct sarcomere molecular signaling in normal atrial sarcomeres

As discussed below, many lines of evidence indicate that homeostatic sarcomere mechanics are required for the maintenance of sinus rhythm (SR), predicting a potential vulnerability in AF and ACM [7], [25], [26], [27], [28], [29]. Atrial sarcomeres express specialized protein isoforms that support chamber-specific function and contribute to unique vulnerabilities. Recent discoveries—especially regarding thick-filament regulation and protein crosstalk—have reshaped understanding of atrial and ventricular sarcomere control mechanisms [16], [30], [31], [32], [33]. We next discuss normal molecular signaling mechanisms with emphasis on those unique to atrial sarcomeres. Despite the need for up-to-date understanding, reviews have limited or no discussion of atrial sarcomere-related mechanisms [8], [34], [35], [36]. These mechanisms have taken on new significance with recent progress, building on early seminal research in identifying small-molecule sarcomere activators and inhibitors that improve cardiac function in cardiac myofilament-related disorders [37], [38], [39], [40].

**Fig. 1:**
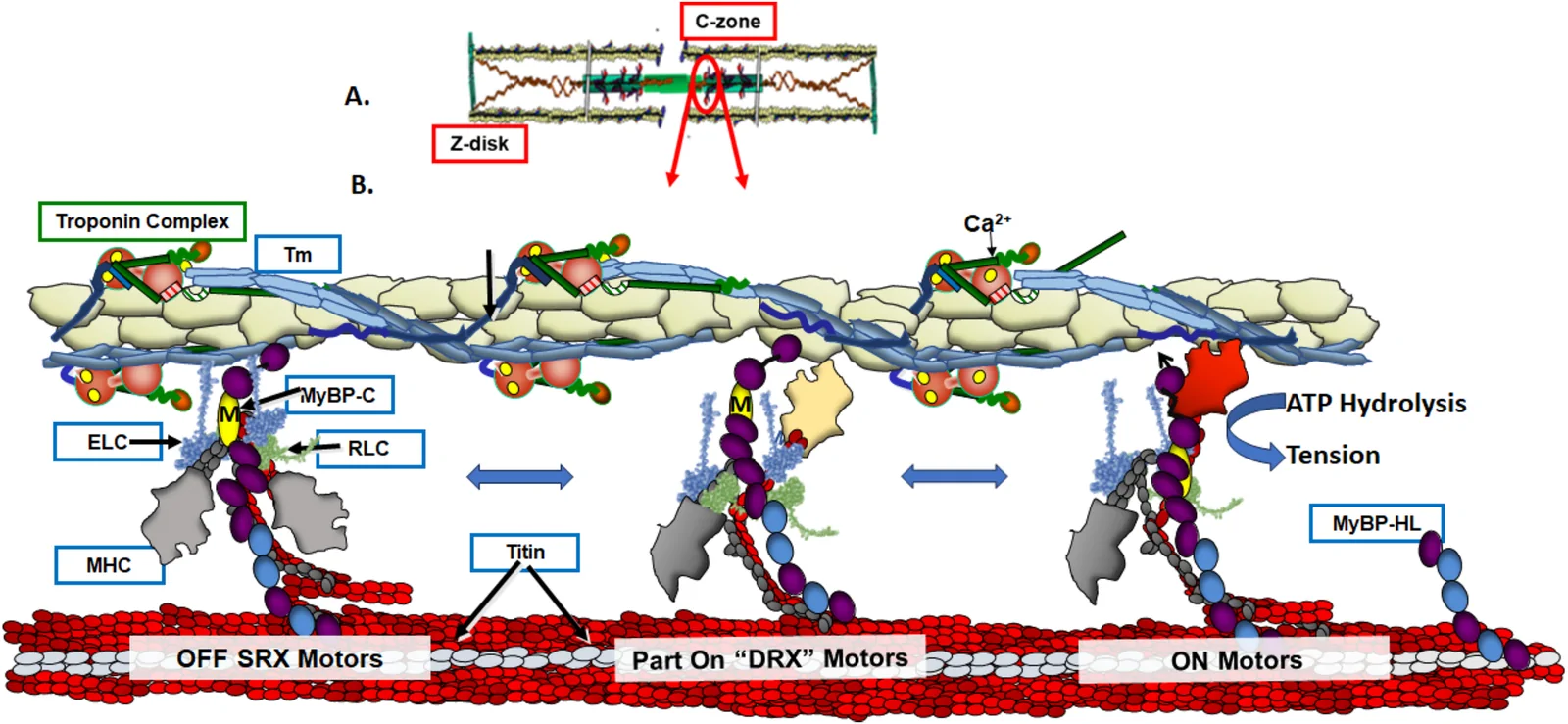
Variations of states of regulatory units (RUs) in the C-zone of atrial sarcomeres. A, Atrial sarcomere showing cardiac myosin binding protein C (cMyBP-C) containing C-zones, which also contain (MyBP-HL) myosin binding protein-HL. Z-disks connect thin filaments and link titin to the M-band. B. C-zone regulatory RUs in a relaxed state with a SRX (grey) motor, with a DRX motor (orange), and with a force generating motor (red), inducing thick filament strain. In the thin filament OFF sate, tropomyosin (Tm), which blocks acto-myosin interactions, is immobilized by strong binding of the cardiac troponin I (cTnI) inhibitory peptide and weak binding of cTnI switch peptide to the cTnC regulatory Ca-binding site, strong binding of the cTnI mobile C-terminal domain and cTnT N-terminus to actin-Tm, and cTnT-C-terminal interactions with cTnC. In early systolic tension, strain modifies the states of N-terminal, middle, and C-terminal domains of cMyBP-C that influence the state of actin-Tn-Tm as well as the movement of DRX motors toward the thin filaments that may bind to TnT. The myosin light chain 2 phosphorylation (MLC2a-P) promotes the strain dependent movement of DRX motors toward the thin filaments. Extension of MLC1a and interactions of DRX motors may interact with the thin filaments, affecting the Tn-Tm inhibition in early systole. ON RUs cooperatively activate near neighbors, giving rise to steep relations between cTnC Ca-binding and tension. See text for further discussion. (For interpretation of the references to color in this figure legend, the reader is referred to the web version of this article.)

Fig. 1 and its legend describe the fundamental control mechanisms in atrial sarcomeres activation and relaxation. These mechanisms involve integrated control of titin, thin, and thick filament proteins (myosin motors, myosin binding protein C (cMyBP-C) and its paralog MyBP-HL (myosin-binding protein H-like)), which is unique in atrial myocytes. These elements operate within the regulatory units (RUs) consisting of actin: Tn (troponin): Tm (tropomyosin): myosin in a 7:1:1:1 ratio [16], [29], [35]. The atrial RUs operate in concert with titin a major determinant of diastolic tension [41], [42]. Compared to ventricular sarcomeres atrial sarcomeres express a relatively high proportion of the longer, more compliant titin N2BA variant [41], [43]^,^ [44], [45], [46], [47]. Specific features of atrial sarcomere regulation are discussed next.

### 2.1 Atrial myofilament proteins and thin filament triggering of atrial sarcomere activation

Activation of thin filaments by Ca^2+^-binding to cTnC is an absolute requirement for release of cardiac sarcomere from inhibition [48]. Variations in TnC-bound Ca^2+^ can determine the number of active RUs participating in the P—V loop in both atria and ventricles. It has been reported that slow skeletal TnI (ssTnI), the fetal/neonatal isoform of TnI, is re-expressed in the adult human atrium [49]. In a population of patients in SR and paroxysmal AF, 60% demonstrated a low expression of slow skeletal TnI (ssTnI), whereas this expression was only 8% of patients in chronic AF [49]. Decreased expression of theTNNI1, the ssTnI gene has also been reported in genomic data bases [50]. We have shown that increased expression of ssTnI in mice hearts induced cardiac protection against multiple cardiac stressors but also increased susceptibility to arrhythmias [51], [52], [53], [54]. The role of re-expression of ssTnI in human atria electrical stability requires further investigation.

An interesting specialization of atrial thin filaments is the presence and a substantial increase in expression of nebulin compared to ventricular myofilaments; both express nebulette [55]. Nebulin extends from the Z-disk to the M-band whereas nebulette a shorter protein extends from the Z-disk along the thin filament (~ 10 actins) [56]. These proteins function in myofilament assembly and myofibrillogenesis, thereby affecting atrial size, and as controllers of sarcomere dynamics by suppression of cross-bridge kinetics [14], [57], [58]. As discussed below there are data indicating a role for these proteins in AF/ACM.

### 2.2 Control of atrial sarcomeres by shifts in the population of MHC isoforms and variations in “sequestered” and “on” myosin motors

A notable sarcomere specialization important in tuning the atrial beat is a predominant expression of α-MHC (~80%) compared to ventricular sarcomeres (~90% β -MHC) [34], [35], [46]. Compared to β-MHCs, α-MHCs have relatively fast actin-cross-bridge kinetics, faster rates of tension development, ejection, and higher peak power generation [59]. An important finding is that a change in the α-MHC population as small as ~10% can change the power output of the sarcomeres by as much as ~50% [59].

Apart from MHC isoform population, an important aspect of the specialized regulation of atrial RUs is a new understanding of the role of thick filaments in RUs [33]. Emerging evidence from biochemical and structural analyses, and cryo-electron microscopy, rooted in the identification of a variable interacting head motif in myosin, has established the existence of subsets of myosin heads engaged in stress-dependent control of sarcomere function (Fig. 3) [29], [60], [61], [62], [63], [64]. One population exists in an “off”, “blocked” super-relaxed state (SRX), with a very low ATPase rate, unable to engage in force production. A second population is myosin heads in an “partially on”, weak, or disordered relaxed state (DRX), which is primed for transfer to a force-generating reaction with actin upon cTnC Ca-binding (Fig. 1) [30], [65]. This thick filament-related mechanism has taken hold as an explanation for altered contractility in physiological states as well as pathologies associated with HF, hypertrophic cardiomyopathy (HCM), and dilated cardiomyopathy (DCM) [62], [63], [66], [67], [68], [69]. As discussed below, there is evidence that concerted interactions among the states of titin, MyBP-C, and atrial myosin light chain 2 (MLC2-a) and ventricular MLC2 (MLC2-v) modify the SRX/DRX ratio.

### 2.3 Control of atrial sarcomeres by shifts in the population and phosphorylation of myosin light chains

Atrial myosin light chains (MLC1-a and MLC2-a), which have specialized functions, are associated with myosin heads and regulate myosin motor function and RU activation. They function in concert with MHCs and other myofilament neighbors in tuning myosin motor force generation and kinetics to ensure atrial homeostasis under the prevailing heart rate, mechanical state, and load [36], [70], [71], [72], [73]. As depicted in Fig. 1, atrial MLCs wrap around and interact non-covalently with the helical C-terminal neck region to regulate the myosin motor; they also have unique N-terminal extensions, which are flexible and longer than their counterparts in skeletal and smooth muscle, which engage in versatile control mechanisms with neighbors [74], [75].

#### 2.3.1 Regulation by atrial myosin light chain 1

The importance of atrial myosin light chain 1 (MLC1-a) in atrial function is underscored by the strong association between atrial fibrillation (AF) and mutations in MYL4, which encodes MLC1-a [76], [77]. A special function of MLC-1 is associated with its N-terminal peptide, which is highly charged, proline-rich, and binds actin, tethering cross-bridges and enhancing thin-filament activation [74], [75], [78], [79], [80]. Structural modeling suggests this ~43-amino-acid extension can span ~90 Å from the thick filament to actin [80]. Functional studies in mouse hearts expressing a ventricular MLC1 variant lacking this N-terminal extension (MLC1-vΔ43) show increased stabilization of myosin in the super-relaxed (SRX) state, supporting a regulatory role for this region myosin [81]. Differences in N-terminal structure likely explain the weaker actin binding of MLC1-a compared with MLC1-v, consistent with the rapid dynamics of atrial contraction [74], [82], [83]. Reconstitution and transgenic studies confirm that the MLC1-a peptide is functionally relevant in vitro and in situ [84], [85]. While MLC1-v is restricted to adult ventricles, MLC1-a expression may be beneficial in failing heats [75], [86]. Notably, phosphorylation of human MLC1-a has not been confirmed by high-resolution mass spectrometry [74], [75]. Overall, evidence indicates that MLC1-a modulates thin- and thick-filament interactions, warranting further investigation in normal atrial physiology, AF/ACM pathogenesis, and therapeutic strategies.

#### 2.3.2 Regulation by atrial and ventricular myosin light chains expressed in atria

MLC2-a, which is normally restricted to adult atria, plays specialized roles in atrial function and is essential for cardiac development during embryogenesis [36], [75]. Transgenic mouse studies expressing ventricular MLC2-v in atria demonstrate that MLC2 isoforms contribute to functional differences between atrial and ventricular muscle, with MLC2-v increasing shortening velocity independent of phosphorylation [87], [88]. This finding is physiologically relevant, as MLC2-v is normally expressed in human atria (~13% in right atrium and ~ 25–30% in left atrium) [74], [89]. This difference may be related to the existence of higher incidence of AF in LA vs. RA [90].

Beyond isoform expression, phosphorylation of MLC2 is a key regulator of atrial function and is altered in AF. Since the initial discovery of MLC2 phosphorylation in the beating heart over 50 years ago, accumulating evidence has established phosphorylation of MLC2-v as a key regulator of atrial and ventricular contractility [27], [36], [71], [72], [91]. Advances in sarcomere biology and high-resolution methods continue to clarify this role. Reported basal phosphorylation levels (~0.2–0.5 mol P/mol MLC2) vary across species, chambers, and sex [91], [92], [93], [94], [95]. Precise measurements have been made in human hearts. Bayne et al. reported a precise determination of basal levels of MLC2 phosphorylation in normal adult male and female human heart samples [75]. Average phosphorylation in MLC-2v was 0.15 in the LV and 0.13 mol P/mol MLC2-v in the RV. Similar levels of 0.19 mol P/mol MLC2-a in LV and 0.16 in the RA were reported. Bayne et al. and Scruggs et al. also reported a precise site of phosphorylation in non-failing human hearts at Ser 15 [75], [96]. MLC2-a phosphorylation is at Ser 22,23. MLC2-v, unlike MLC2-a, demonstrates a deamidation post-translational modification (PTM) located in the N-terminal region at N13, which provides a charge group in addition to the phosphorylation sites. This additional charge mimics the charge effect of the two-site MLC2-a phosphorylation. Although these specific regulatory mechanisms in atrial MLC2-v and MLC2-a appear important in understanding normal and abnormal atrial function, precise determinations of their significance remain unclear [75].

MLC2 phosphorylation is regulated by complex kinase–phosphatase signaling, including cardiac myosin light chain kinase (cMLCK) and myosin light chain phosphatases. A variety of approaches in studies of ventricular control mechanisms at various levels of organization support the important regulatory role of phosphorylated MLCs (MLC2-v-(P) and MLC2-a-(P)) in atrial tissue and provide mechanistic insights. Complex signaling involving clusters of regulatory factors, kinases, and phosphatases potentially regulates MLC2 phosphorylation and dephosphorylation. There is a tissue-specific cardiac myosin light chain kinase (cMLCK) with a unique N-terminal structure and regulated by Ca^2+^-calmodulin that is expressed in both the atrium and ventricle [97], [98]. A specific regulatory subunit of protein phosphatase 1 (PPP1R12C (protein phosphatase 1 regulatory subunit 12C)), has emerged as a regulator of MLC2a-(P) [27], [99], [100], [101]. Collectively, these studies support a central role for MLC2 isoforms and their phosphorylation in atrial mechanics, while highlighting the need for direct studies in atrial health and disease.

### 2.4 Sarcomere control by interactions of cMyBP-C and titin with thick and thin filaments

As summarized in Table 1, linkage of induction of AF in DCM and HCM with disease genes encoding MYBPC3, and in DCM disease genes encoding TTN provides a strong rationale for understanding how cMyBP-C and titin integrate their activity to contribute to atrial mechanical/electrical stability and ACM [102], [103], [104]. As shown in Fig. 1, atrial myocytes express cMyBP-C, which interacts with myosin, titin, and thin-filament proteins, like ventricular muscle, though atrial cells use additional mechanisms to control cMyBP-C abundance. In the relaxed (OFF) state, C-terminal cMyBP-C domains bind myosin tails and titin, forming a 43 Å periodicity [105], [106], [107], [108], [109]^,^ [110], [111]. De-phosphorylated cMyBP-C also binds myosin motors, stabilizing the OFF state, while a minority of N-terminal segments contact thin filaments [108]. With Ca^2+^ activation, cross-bridge interactions displace N-terminal cMyBP-C from thin filaments and alters myosin kinetics. Phosphorylation, especially by PKA, regulates the flexibility of these interactions [108]. Phosphorylation releases N-terminal binding to actin–tropomyosin, promotes myosin motor movement toward thin filaments, increases shortening velocity, and enhances Ca^2+^-sensitivity and cross-bridge kinetics [106], [112]. Titin-based mechano-sensing also contributes, though its effect is smaller in atrial versus ventricular myocytes.

**Table 1:** Summary of sarcomere remodeling changes and related molecular findings in genetic cardiomyopathies.

Ref.	1st author	Gene (cardiomyopathy)	Model, chronicity	Sarcomere remodeling changes	Other major molecular findings
[106]	Ahlberg, G	TTN (DCM, ACM)	Human, whole exome sequencing	Titin-truncating variants (TTNTV) associated with early onset AF (EOAF), compromised assembly of the sarcomere	Longer PR interval on EKG, significant atrial fibrosis
[107]	Choi, Sh	TTN, KCNN3, PRRX1, PITX2, NEURL1, SOX5, ZFHX3 (ACM)	Human, whole genome sequencing	Titin loss of function (LOF) variants are associated with EOAF	GWAS Results in EOAF: KCNN3, PRRX1, PITX2, NEURL1, SOX5, ZHFX3
[109]	Rosas, PC	MYBPC3 (HFpEF)	Mouse, MYBP-C activating/inhibiting	cMyBP-C(t2SA) phosphorylation deficient mice have impairment of relaxation and HFpEF	Constitutively activated cMyBP-C mice (t3SD) have enhanced relaxation in part due to faster rate of cross-bridge detachment
[111]	Ponnam, S	MYBPC3 (HCM)	In Vitro binding assays, in situ structural measurements in intact heart cells	PKA-dependent phosphorylation of cMyBP-C N-terminus antagonizes PKCe phosphorylation, controls interaction with thick and thin filaments	RSK2 phosphorylation of cMyBP-C accelerates PKA phosphorylation
[112]	Chandler, J	MYBPC3 (HCM)	Rat Neonatal CMs, In Vitro, FRET assays	in relaxed sarcomeres, C-terminal and mid-regional domains of dephosphorylated cMyBP-C bind to the myosin motors, promoting the OFF state	With Ca^2+^-activation, cross-bridge interactions impede the binding of N-cMyBP-C to the thin filaments; interactions with S1-S2 regions are now tethered with myosin motors, affecting cross-bridge kinetics
[113]	Gilbert, R	MYBPC3 (HCM)	In Vitro, transfection of truncated MyBP-C mutants in skeletal muscle myoblasts	The myosin binding domain of MyBP-C is not required for A-band insertion, but motifs VII to IX are required for binding	–
[114]	Freiburg, A	MYBPC3 (HCM), Titin (DCM, ACM)	Rabbit, isolated fast-skeletal MyBP-C	C-terminal domains (C8-C10) of cMyBP-C bind to the myosin tail regions and C-regions of titin, giving rise to a 43 Å-periodicity	–
[115]	Belknap, B	MYBPC3 (HCM)	Rabbit, isolated fast-skeletal MyBP-C	N-terminal cMyBP-C domains are sufficient to inhibit steady-state actin-activated ATP hydrolysis.	N-terminal domains of human cMyBP-C produce biphasic activating and inhibitory effects on thin filament-activated ATPase activity when native cardiac thin filaments are used to activate myosin ATPase activity
[116]	Barefield, DY	MYBPC3 (HCM), MYBPH (conduction), MYBPHL (conduction)	Mouse, atria, Mybphl−/−	Localization of MYBP-HL in the sarcomere tracks with the 43 nm banding seen with MyBP-C	Complete loss of MyBP-HL results in doubling of MyBP-C; MyBP-HL occupies one additional 43 nm repeat toward the center of the atrial sarcomere
[117]	Colson, BA	MYBPC3 (HCM), TNNI3	Mouse, skinned trabeculae	cMyBP-C phosphorylation relieves a constraint on cross-bridges, increasing the proximity of myosin to actin binding sites	Constitutive activation of cMyBP-C phosphorylation site protects hearts from hypertrophy and systolic dysfunction
[118]	Mamidi, R	MYBPC3 (HCM)	Mouse, skinned ventricular prep	Decreased cMyBP-C phosphorylation results in impaired modulation of length-dependent changes in cross bridge kinetics	cMyBP-C phospho-ablation significantly blunts the magnitude of cooperative cross-bridge recruitment and abolishes PKA-mediated accelerations in the rate of cross-bridge recruitment
[119]	Kumar, M	MYBPC3 (HCM), TNNI3	Mouse, PKA site on cMyBP-C activated or ablated	PKA-mediated phosphorylation of cMyBP-C and cTnI each independently enhance myofilament length-dependent activation of the sarcomere	–

Atrial tissue uniquely expresses MyBP-HL, a shorter paralog like the C-terminus of cMyBP-C, at about half the abundance of cMyBP-C. MyBP-HL occupies binding sites normally used by cMyBP-C; deleting MyBP-HL increases cMyBP-C levels to ventricular-like amounts and accelerates myofibril shortening [35], [113]. Thus, MyBP-HL modulates atrial contractile kinetics by controlling cMyBP-C availability and influencing Ca-sensitivity, cross-bridge behavior, and length-dependent activation [114], [115], [116]. MyBP-HL also appears to affect electrical stability. Human MYBPHL truncations are associated with DCM, atrial enlargement, and arrhythmias. MYBPHL-null mice show atrial and ventricular arrhythmias linked to conduction defects and abnormal Ca-handling [117]. Further work is needed to define underlying molecular mechanisms.

## 3 Interplay of atrial sarcomere activity and regulation in the physiological atrial pressure-volume relations

**Fig. 2:**
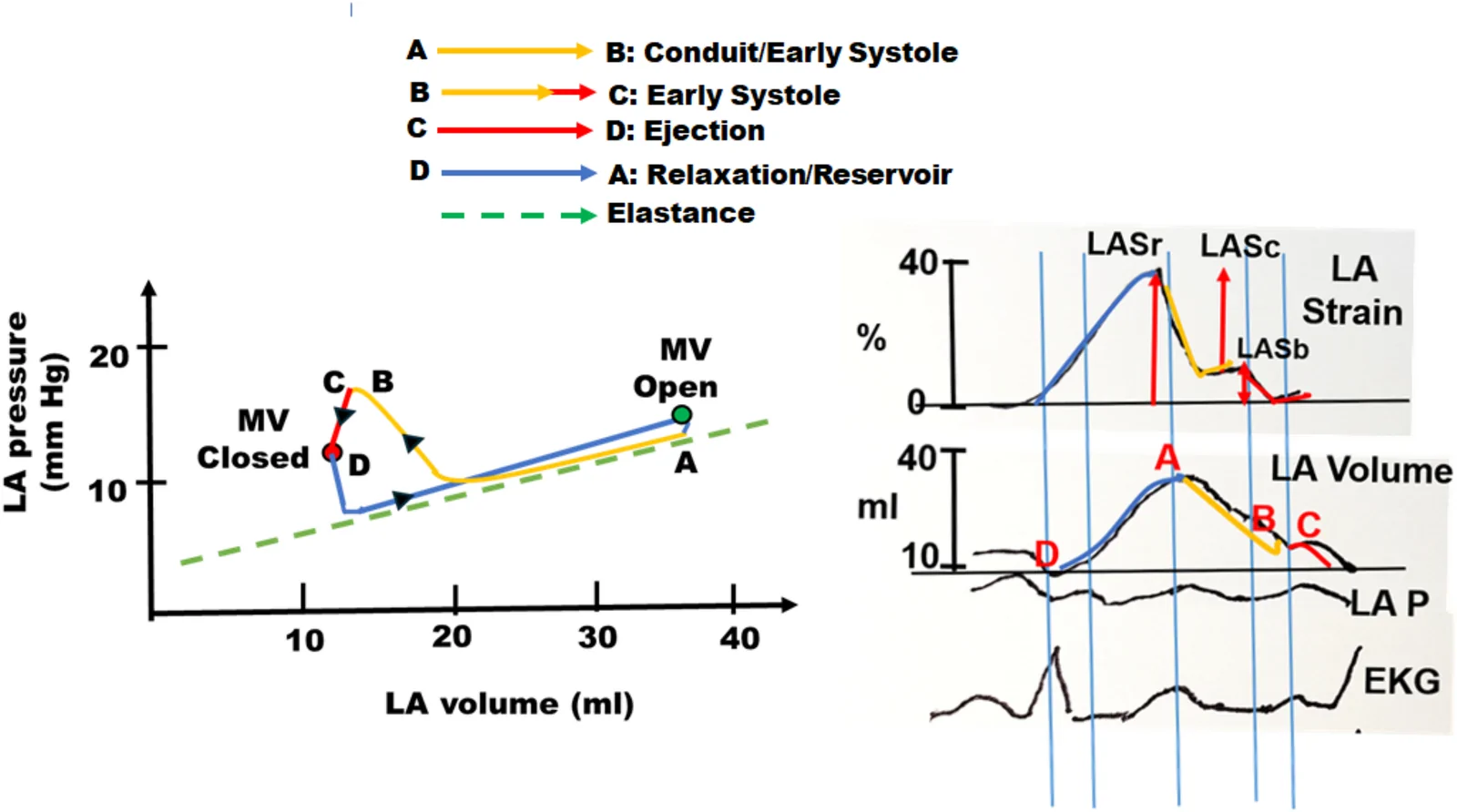
Left panel. Left atrial pressure-volume (P-V) loop. Colored lines denoting reservoir (filling of the atrial chamber), conduit (transfer of volume to left ventricle), and ejection phases, in which atrial contractions add a “booster” volume of blood (~15–30%) to the ventricles within ~100 msec. The slope of the dashed line indicates elastance during filling (Adapted from Wright et al. [118]). See Fig. 3 and text for the state of sarcomere RUs in each of the phases. Right panel. Different elements of LA strain measurement by speckle tracking echocardiography shown with time dependent changes in LA volume, pressure, and the EKG. LASr (LA reservoir strain); LASc (LA conduit strain) and LASb (LA booster strain). Note that LV strain is most prominent during the reservoir phase.

Fig. 2 shows the time course of changes in LA pressure (P) and LA volume (V). and a depiction of the P—V loop, based on data reported by Wright et al. [118]. Each measure of LA function provides a view of the phases, which include a reservoir phase in which LA filling occurs during LV systole by pulmonary vein flows. The slope of the dashed line in the P—V loop indicates the elastance (mm Hg/ml) of the reservoir phase reflecting the atrial stiffness during filling. A conduit phase follows in which there is passive LA flow filling the LV. Finally, there is a booster-pump phase in which the LA actively ejects blood into the LV. Also shown Fig. 2 is LA strain (LASr, reservoir strain; LASc, conduit strain, and LASb, booster strain) determined by speckle tracking echocardiography in a young healthy male by O'Neill et al. [119]. Investigations employing speckle tracking echocardiography have advanced and provide a quantification of atrial function in the pressure volume loop. This overt indication of atrial deformation and reservoir function are rooted in sarcomere activity during the pressure volume loop affecting atrial elastance and contractile state [120], [121], [122]. Strain determinations indicate chamber deformation during the cycle and indicate that strain is at its peak during the reservoir phase. As emphasized in the literature, speckle tracking echocardiography is especially valuable in the assessment of ACM.

(Adapted from O'Neill et al. [119].)

**Fig. 3:**
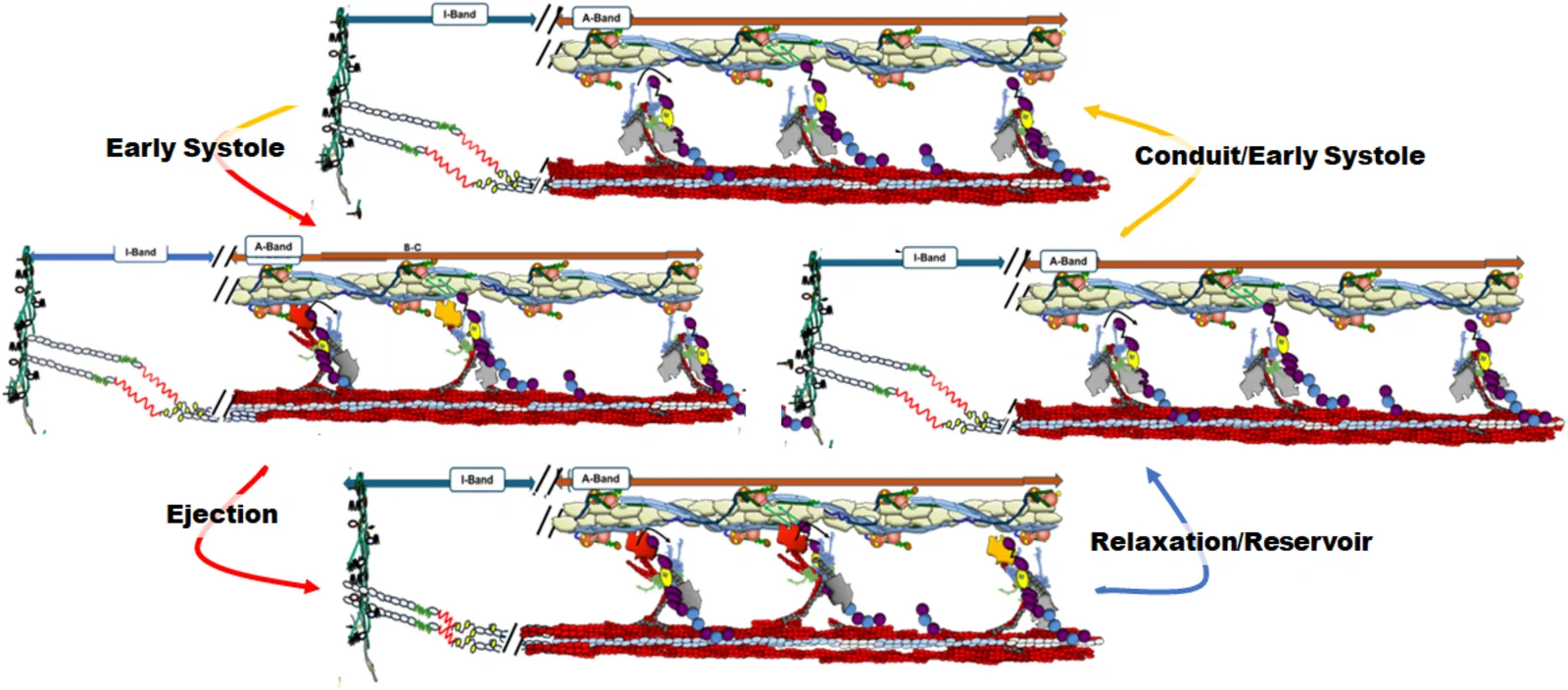
Atrial regulatory units (RUs) in the C-zone of atrial sarcomeres in the transition of phases of the pressure-volume loop. Reservoir relaxed RUs are shown with Off, SRX motors which continues in the conduit phase transitioning to early systole with some motors in the partially on DRX state and On states. More motors enter into the On state promoting ejection. As sarcomere activation wanes there is a transition to the relaxed state/reservoir phase completing the cycle.

Unlike the case in ventricular sarcomeres, investigations in atrial sarcomeres have not investigated cellular and molecular dynamics employing molecular probes, X-ray diffraction, and cryo-electron microscopy. In view of the likelihood of similar fundamental mechanisms in atria and ventricular sarcomeres, we have relied on these determinations to produce a confluence of data allowing a testable hypothesis for the state of sarcomere proteins in relations between atrial pressure and volume as well tracings of strain [31], [32]. Fig. 3 indicates states of the sarcomere RUs during the phases of LA function. During the passive stage, with filling of the atrial reservoir and conduit flow up to atrial systole with ejection into the ventricular chamber, force-generating myosin motors are blocked from reacting with the thin filaments. In these stages, at physiological temperatures, evidence indicates that in relaxed sarcomeres with low thick filament stress, the myosin motors are predominantly in the SRX state (Fig. 2, Fig. 3) [123], [124]. However, during this phase variations in sarcomere length and titin compliance can modify thin filament signaling determining the tension response triggered by Ca^2+^ binding to cTnC. For example, changes in titin stretch and compliance in relaxed sarcomeres have been shown to modulate interactions between cMyBP-C and thin filaments modulating cTnI-cTnC interactions and the state of the thin filament in anticipation of Ca^2+^ release [125], [126]. Moreover, length dependent mechano-signaling can modulate the SRX/DRX ratio by mechanisms involving titin, cMyBP-C, and myosin light chains (MLC) [31], [32], [65], [127], [128]. Myosin motors in the C-region, which are largely responsible for force generation and shortening, are subject to control by this mechano-signaling with variable gain. This gain is influenced by increased VR, leading to an increase in atrial volume; there is an increase in the stretch of titin beyond that occurring in the previous beat. This stretch leads to amplification of the series of reactions described above, resulting in a length-dependent activation in the context of the Frank-Starling relation [125], [126], [129].

The ejection phase of atrial function is triggered with release of Ca^2+^ (Fig. 3). Sarcomeres are released from inhibition, tension increases and contraction ejects the booster volume into LV. A novel perspective is that increased thick filament stress-dependent mechano-sensing stress is a requirement to induce variations in SRX/DRX ratios that control thick filament motor availability for force generation with thin filament activation [31], [32], [130]. Although titin determines stiffness with increases in filling, atria express a more compliant isoform of titin, not likely to induce as significant a stress as in ventricular tissue [44]. It is more likely that the mechano-dependent transition from SRX to DRX motors sets out with the low tension induced early in atrial systole. Fig. 2 depicts RUs in the early response to Ca^2+^ with illustrations of transitions of the thin filaments in the transition from diastole to systole [16], [131], [132]. As Ca^2+^ rises in an early phase of atrial systole, “sentinel” D-zone force-generating myosin motors react with the thin filaments and induce stress on the thick filaments, promoting C-zone force generation [65], [123], [133]. C-zone thick filament proteins, cMyBP-C and MLC2a, sense this stress and promote the transition of SRX motors to DRX motors ready to enter a force-generating interaction with the thin filaments [31], [32], [127], [128]. Moreover, it is possible in early systole that there is an associated modulation of thin filaments state by concerted interactions of cMyBP-C and DRX motors with the thin filaments, affecting the Tn-Tm on state, thereby enhancing the response to the increasing systolic Ca^2+^ [125], [134], [135]. As shown in Fig. 1, extensions of MLC1 may also affect the state of activation of the thin filaments and possibly tether myosin motors [79], [84]. As Ca^2+^ continues to rise, RUs are released from inhibition and C-region motors are recruited to enter the force-generating cycle, generating tension that induces cooperative interactions among regulatory units along the strands of the thick and thin filaments, and, as shown in Fig. 2, eject a booster volume of blood into the ventricular chamber [132], [136]. Return of the atrial P—V relation begins as the Ca^2+^ transient wanes, the thin filament reverts to an inhibited state, and tension and thick filament stress fall, promoting the return of myosin motors to the SRX state (Fig. 3) [61].

**Fig. 4:**
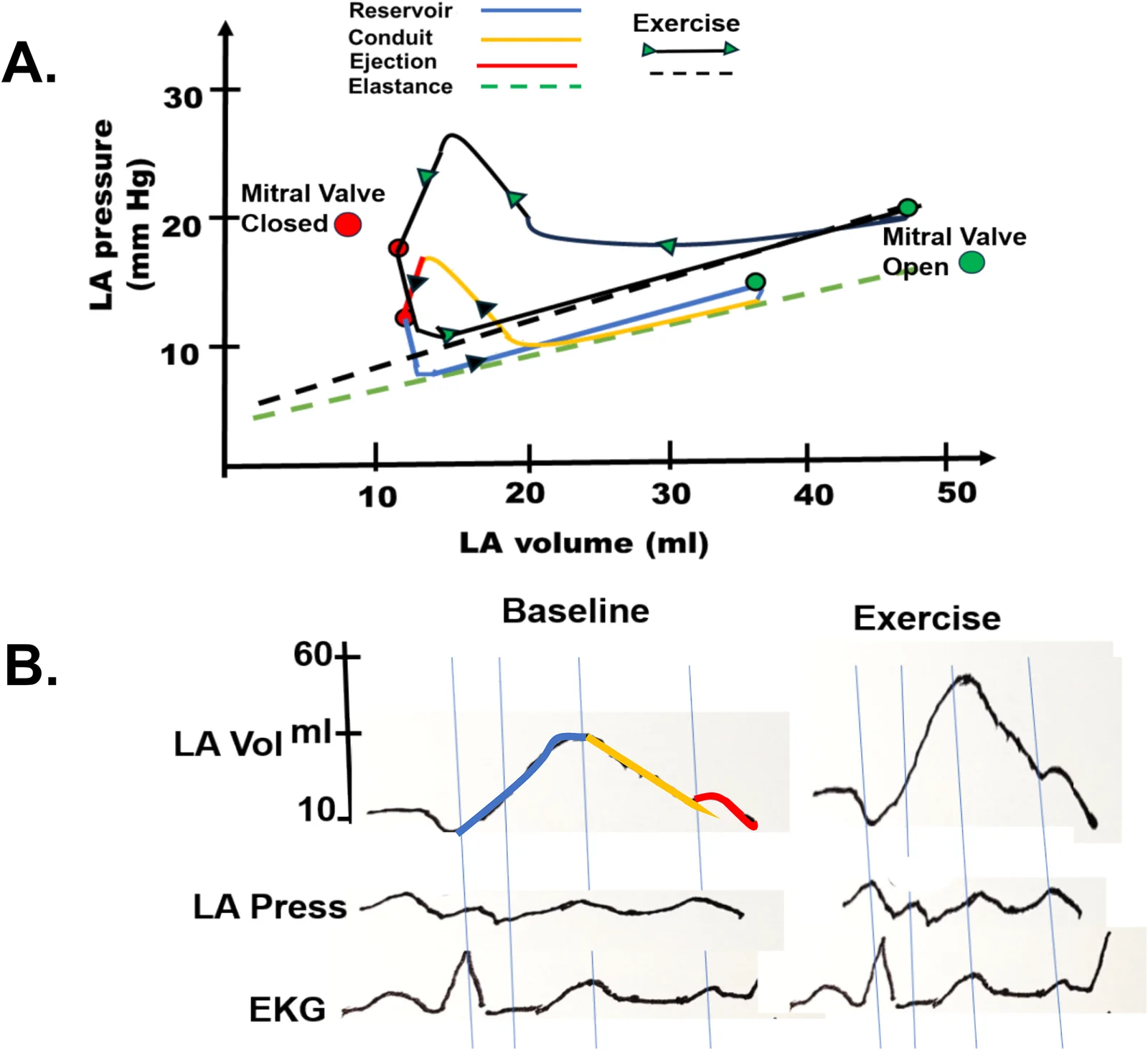
Phases of the left atrial pressure-volume (P-V) in a basal state (colored lines) and in exercise (solid black line). The slope of the dashed lines indicates the elastance, which is increased in exercise, indicating its reciprocal, compliance, declines in exercise together with enhanced kinetics of the phases. See text and Fig. 2 legend for further description. Depiction of the P-V loops is based on human data reported by Wright et al. [118]. (For interpretation of the references to color in this figure legend, the reader is referred to the web version of this article.)

Each of these atrial sarcomere control mechanisms is subject to an array of biochemical/mechano-signaling, PTMs, and influences of the micro-environment of the atrial myocytes. Fig. 4 illustrates an example of modulation of the atrial pressure-volume relations in exercise. With exercise, reservoir volume increases with an increased elastance and little change in conduit volume. With adrenergic activation sarcomere dynamics and Ca^2+^ flows are tuned to the increase heart rate [118].

## 4 Genetic and acquired AF/ACM

**Fig. 5:**
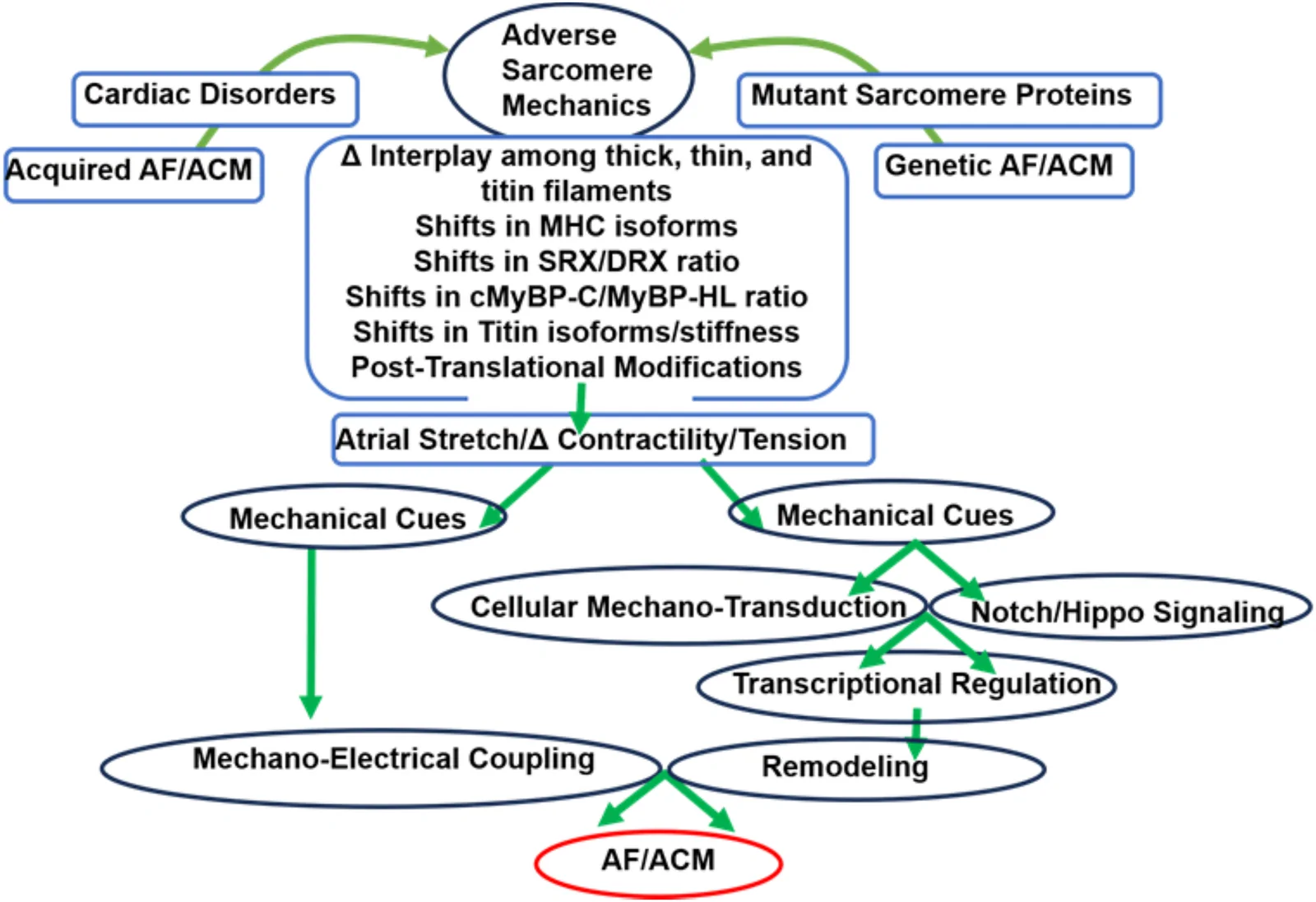
Scheme illustrating shared downstream effects of adverse sarcomere function in genetic and acquired atrial cardiomyopathy (ACM). Genetic ACM directly causes ACM by inducing a transition from homeostatic sarcomere functional flexibility. to persistent mechanical and mechano-signaling dysfunction leading to adverse mechano-transduction/remodeling. The result is induction of altered atrial stretch/contractility that generate mechanical cues leading to alterations in mechano-electrical coupling and mechano-transduction/transcriptional regulation/remodeling. See Fig. 6 and the text for further discussion.

**Fig. 6:**
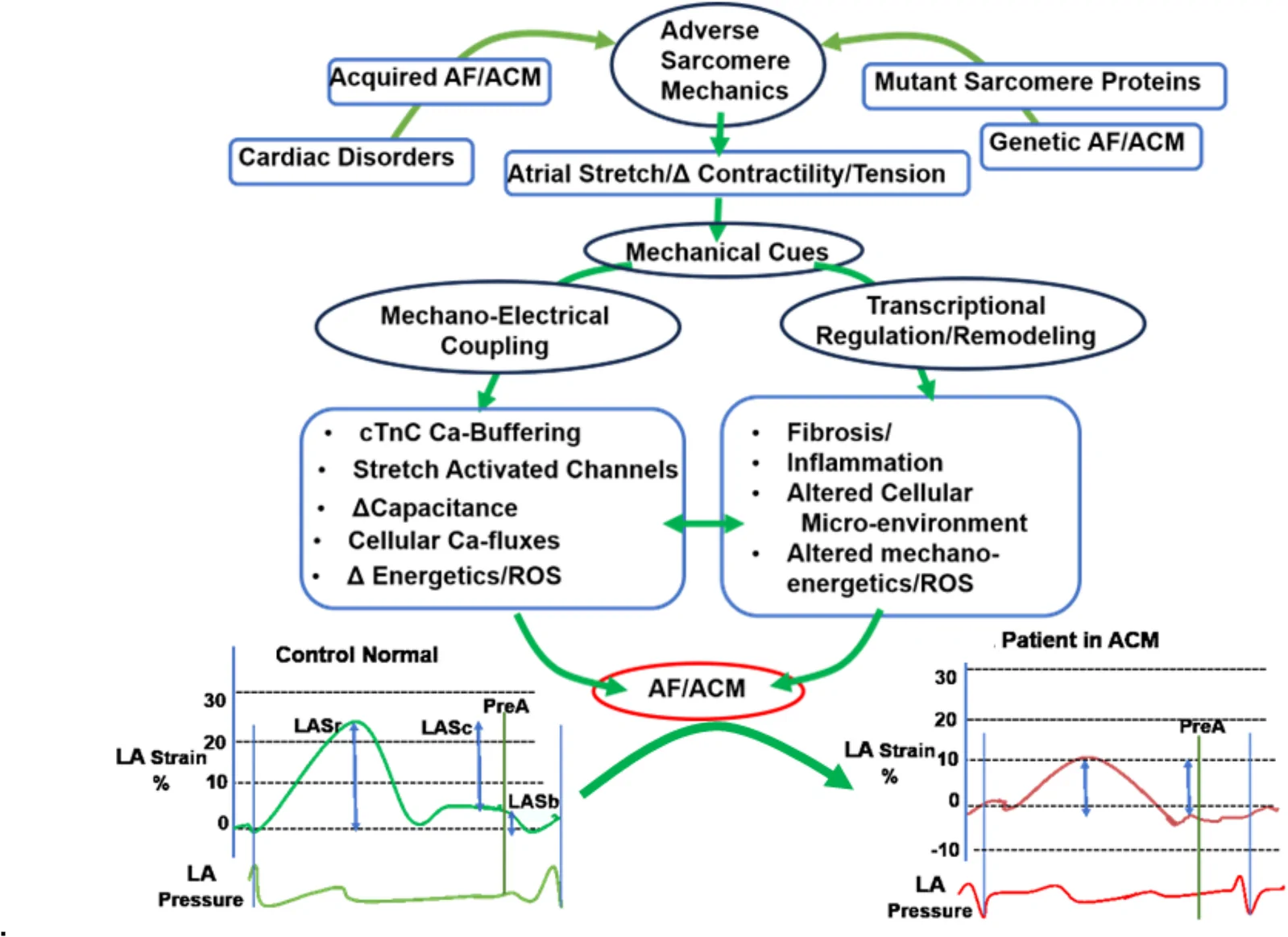
Scheme summarizing roles of adverse sarcomere function inducing altered atrial stretch/contractility that generate mechanical cues leading to alterations in the elements of mechano-electrical coupling and mechano-transduction/transcriptional regulation/remodeling. The response to this pathological process is shown at the bottom of the figure by data redrawn from O'Neill et al. comparing LA strain in control individual and in a patient with ACM [119]. See text for further discussion.

As summarized in Table 1, AF/ACM associated with adverse mechano-signaling via altered sarcomere function can be direct in the case of genetic cardiomyopathies linked to mutations in myofilament proteins. In contrast, indirect AF/ACM may be associated with acquired sarcomere hypo- or hyper-contractility arising from cardiac disorders. After a broad discussion of the prevalence of both genetic and acquired AF/ACM, in the following sections we review evidence for mechanisms that occur downstream of impaired sarcomere function promoting AF/ACM (see Fig. 5, Fig. 6).

### 4.1 Genetic AF/ACM

Data from studies on genetic AF/ACM provide evidence that modifications in sarcomeres whether derived from direct or indirect mechanisms induce a functional inflexibility leading to ACM. As discussed more thoroughly in later sections, expression of disease-causing variants inducing adverse signaling from the sarcomeres promote fibrosis, inflammation, metabolic instability, oxidative stress, endoplasmic reticulum stresses, and adverse neuro-humoral signaling [137], [138], [139], [140], [141]. As summarized in Table 1, a common outcome is electrical instabilities, sudden death, ACM and AF [142]. In HCM, which is the most common familial cardiomyopathy, AF is the most common sustained arrhythmia. In patients with HCM, the occurrence of AF is 20–30%, whereas the occurrence is ~1% of the general population [143]. Incidence varies with age and ranges from ~20- ~ 40%. Comparisons of similar age groups indicate that the occurrence of AF in HCM patients is 4–6-fold greater than in other types of cardiomyopathies [144], [145]^,^ [146]. Guttman et al. reported an overall prevalence of 22.45% for thromboembolism in HCM patients with AF [147].

The prevalence of familial DCM in a large diverse patient population with idiopathic DCM was reported to be 29.7% [148]. Among patients with HCM, restrictive HCM, and DCM the application of catheter ablation in the ER for AF was associated with 29%, 43%, and 19% respectively [145]. There is also abundant evidence in genetic DCM that altered signaling from sarcomeric proteins promotes ACM. An important example is truncating mutants (TTNtv) of the giant atrial protein, titin, which is the most common cause of DCM with a higher risk of arrhythmia and very early onset AF [149] [150]. Patients harboring TTNtv demonstrate risk factors for developing persistent AF [149]. In summary, the data reported above support the need for more in depth understanding of molecular mechanisms in normal and vulnerable atrial sarcomere function in genetic ACM.

### 4.2 Acquired AF/ACM

Whereas there is a straight-forward linkage between signaling mechanisms in sarcomeric proteins and ACM via expression of variants of genes expressing mutants that cause hypo- or hyper-contractility, the role of sarcomere function in the pathology of acquired ACM is indirect. Data indicate that there is an indirect route in cardiac disorders via pathological processes like those signaled by sarcomere abnormalities in HCM and DCM. An explicit review of the common mechanisms in genetic and acquired cardiac disorders has been reported with emphasis on hypertension as a factor promoting post-translational modifications of myofilament proteins thereby affecting sarcomere response to Ca^2+^ in manner like HCM [151].

Other cardiac and related disorders may lead to indirect adverse modifications in sarcomere function leading to AF/ACM. These include roles, for example, of congestive heart failure (CHF) and of disorders, such as diabetes, myocarditis, and obesity [9], [152]. Schmiegelow et al. reported the presence of AF at 9.6% in a population of 2627 patients with heart failure (HF) due to a wide range of non-genetic causes [153]. Myofilaments isolated from failing human heart samples have consistently demonstrated persistent modifications in tension, shortening, and the Frank-Starling relation mimicking the adverse effects induced by HCM and dilated cardiomyopathy (DCM) [19], [154]. In acquired HF, for example, diastolic dysfunction and age were positively correlated with AF in patients with heart failure with preserved ejection fraction (HFpEF), two-thirds of whom had AF [155].

Apart from the strong relations between sarcomere function in acquired and genetic AF/ACM, there is additional evidence is provided by multiple studies in membrane-free myofibrillar preparations isolated from atrial samples from humans in AF compared to controls in SR [26], [27], [28], [46], [156], [157]. These high-fidelity in vitro investigations demonstrate that control of tension and dynamics is chronically altered in AF/ACM. Moreover, specific modifications in sarcomere proteins identified in human atria in AF can induce AF when introduced into the atria of wild-type murine models [27], [157]. A significant modification associated with AF/ACM is altered myofilament protein phosphorylation, which is summarized in Table 2 and discussed for each of the proteins below. Modifications of atrial sarcomere-related control mechanisms in situ also demonstrate induction of AF [27], [29], [157]. Myocytes isolated from the RA of dogs stressed by short-term atrial tachycardia showed a significant decrease in atrial and sarcomere contractility, with no change in measures of sarcoplasmic reticulum function [26]. Moreover, some studies have integrated altered specialized membrane and sarcomere mechanisms in atria in simulations of SR and AF [158], [159]. In summary, these data emphasize the need to understand atrial mechanics at the molecular level to better understand pathological mechanisms in acquired AFM and to inform therapeutic approaches. We next discuss specific modifications in atrial sarcomere proteins that potentially signal AF/ACM. This is followed by consideration of the pathways by which these modifications lead to atrial pathology.

**Table 2:** Summary of changes in sarcomere protein phosphorylation associated with various atrial samples from humans and models exhibiting AF/ACM.

Ref.	1st author	Model/atrial preparation	Changes in atrial sarcomere mechanics with AF/ACM	Changes in protein phosphorylation and other changes with AF/ACM
[27]	Perike	Atrial samples from humans in SR and chronic AF	N/A	MLC2a-P (−)
				Increase expression PP1R12C
[27]	Perike	Mice expressing PP1R12C permeabilized fiber bundles and single myocytes	Decrease in tension, power and rate of shortening	MLC2a-P (−)
[159]	Belus	Myofibril size preparations from right atrium of humans in SR and AF	Reduced rates of active tension and rates of rise and fall in tension. Increase in Ca^2+^-sensitivity and passive tension	cMyBP-C-P (+)
				MLC2a-P (+)
				cTnI-P (NC)
				cTnT-P (+)
				Increased expression of MHC-β. Increased expression of titinN2B
[28]	Eiras	Permeabilized myocytes from right atrium of humans in SR and AF with dilatation	Reduced rates of cross-bridge kinetics	cTnT-P (+)
				Increased expression of MHC-β
[161]	El-Armouche	Samples from right atrium of humans in SR and chronic AF	N/A	cMyBP-C-S282-P (−)
				cTnI-P (NC) Increased PP1 and 2APP
				Phospholamban-P (+)
[25]	Wakili	Dog tachypacing/permeabilized fiber bundles	Decrease in Cross-bridge kinetics	cMyBP-C-P (−)
				MLC2a-P (−)
				cTnI-P (NC)
				PKA regulatory proteins (−)
				PPI (+)
				CamKinase (+)
[166]	Greiser	Goats with atrio-ventricular block Atrial fiber bundles	Decrease in fractional shortening	cTnI-P (−)
[166]	Greiser	Goats with burst tachypacing Atrial fiber bundles	Decrease in fractional shortening	cTnI-P (NC)

## 5 Pathogenic atrial sarcomere mechanics as an element in the pathology of AF/ACM

### 5.1 Adverse thin filament modifications in AF and ACM

All three Tn components have potential roles in AF/ACM. AF in patients harboring mutations in cTnT linked to hypertrophic cardiomyopathy points to an important role of atrial cTnT and thin filaments in the electrical stability of the heart [142]. Increased phosphorylation of atrial cTnT, which may also contribute to AFM and AF, has been reported to occur in myofilaments isolated from AF patients compared to controls [28], [156]. Phosphorylation of cTnT is likely to be an important contributor to the reduction in maximum tension in the AF samples. We have reported that phosphorylation of cTnT by protein kinase C in ventricular sarcomeres decreases maximum tension [160]. Cardiac disorders are known to be correlated with changes in atrial thin filament phosphorylation some of which have also been reported to occur with AF and likely important in ACM (Table 2) [28], [46], [156], [161]. As discussed below effects of proteolysis of thin filament components is a documented contributor to altered mechano-electrical signaling and induction of persistent AF.

As discussed above expression of atrial nebulin and nebulette, which interact with thin filament proteins may be associated with AF. Evidence indicates that the functional states of these proteins include missense mutations in nebulette associated with AF as well as in reduced nebulin expression in samples from AF patients compared to controls. Genome wide association studies identified genetic loci in the nebulette gene, NEBL, associated with AF in a large patient population, a finding considered relevant to AF susceptibility [50]. Upregulation of NRAP (nebulin related anchoring proteins) was found in atrial samples from patients in AF, indicating a modulation of nebulin linked to AF. NRAP, which is present in myofibril precursors, is a controller of myofibril assembly, which may be a significant factor in controlling atrial size [50].

### 5.2 Adverse effects of shifts in the population of myosin motors, MyBP-C/MyBP-HL, and titin isoforms and cMyBP-C phosphorylation in AF and ACM

In human AF, there is a decrease in expression of the fast α-MHC in atrial cells with a significant increase in the β-MHC population from ~20% to ~40% [28], [156]. As indicated above small changes in the α-MHC population induce changes in the power output sarcomeres significantly reducing atrial contractility and signaling adverse remodeling [27], [59]. As discussed above, beyond a modification in atrial dynamics and power shifts in the MHC isoform population would be expected to alter the SRX/DRX ratio by an effect on the gain in mechano-sensing. As discussed below, there is evidence that the states of MLC2a and MLC2-v modify the SRX/DRX ratio. Although not investigated adverse modifications in ratio of MyBP-HL/cMyBP-C represent an important point of vulnerability in AF and ACM. Functional stability of cMyBP-C requires variation in post-translational modification by a variety of kinases, with a major role of protein kinase A (PKA)-dependent phosphorylation of a unique region at the cMyBP-C N-terminus [108]. As summarized in Table 2, except for one study, a persistent decrease in cMyBP-C phosphorylation has been reported in acute animal models of AF, as well as in chronic human AF [55], [58], [80]. Phosphorylation of cTnI has also been determined to be significantly reduced in an AF goat model [162]. S-glutathionylation of titin, cMyBP-C, and cTnI also affect their function and needs to be evaluated as an effect of oxidative stress in AF/ACM [105].

Titin, a controller of diastolic function, plays a significant role in the homeostasis of the atrial sarcomeres, which spend ~80% of [59] their time in a variable relaxed state during the heartbeat. As summarized in Table 1, evidence for this role is that prevalent truncating mutants of titin are a cause of electrical instability in non-ischemic DCM [149]. The abundance of titin in ventricular and atrial muscle appears to be the same [103], but compared to ventricular myocardium, atrial myocardium expresses a higher proportion of the longer, more compliant N2BA variant, resulting in relatively lower passive stiffness important for rapid filling of the atrial compartment [44], [45], [46] [47]. In AF, there are shifts in the isoform population, reducing the population of NB2A in myofibrils, inducing adverse passive tension [44], [45], [46]. Moreover, altered stiffness in titin has been reported to affect the Frank-Starling relation [41], [43]. We have reported evidence in ventricular myofilaments that this signal involves sensing titin stretch by interactions with myosin tails in the thick filament and a complex interplay among MHC, SRX/DRX motors, cMyBP-C, MLC2, the state of TnT, and the TnI-TnC interaction [125], [135].

### 5.3 Adverse modifications of control by MLC2-v and MLC2-a in AF and ACM

Flexible modulation of atrial MLC2 isoform populations and N-terminal phosphorylation is essential for atrial homeostasis; both are altered in AF. Regulation of MLC2 isoform expression appears important for atrial homeostasis, as MLC2-v levels increase under pressure overload and in animal models of AF [89], [163]^,^ [164]. Conversely, MLC2-a is re-expressed in ventricles during cardiac stress, with effects that may be adaptive or maladaptive [64]. Early studies showed increased atrial MLC2-v expression under pressure overload and in hypertensive rat hearts, findings later confirmed in a porcine AF model with elevated atrial MLC2-v compared to controls [89], [163], [164].

As discussed above, cardiac myosin light chain kinase and the PP1 regulatory subunit PP1R12C, which is elevated in human AF, have emerged as key regulators of MLC2 phosphorylation [27], [98], [99], [100], [101]. We recently demonstrated reduced MLC2-a phosphorylation in right atrial samples from AF patients compared to SR controls, highlighting a potentially important therapeutic mechanism. In a mouse model reproducing this defect, we observed atrial enlargement, reduced strain, and increased AF susceptibility. These studies identified an association of a reduction in MLC2a phosphorylation with impaired sarcomere power generation as a major contributor to hypo-contractility [27]. MLC2 phosphorylation also modulates stretch-dependent myofilament signaling, influencing length-dependent activation and the Frank–Starling response force [165]. These findings underscore the importance of MLC2 phosphorylation and sarcomere remodeling in AF/ACM.

## 6 A role of altered sarcomere function in adverse mechano-transduction/remodeling in AF/ACM

### 6.1 Sarcomere signaling in mechano-transduction/remodel

Fig. 5 illustrates evidence for the mechanical cues generated by the adverse sarcomere function described above. These cues are likely to engage pathways promoting altered mechano-electrical coupling and altered transcriptional regulation/remodeling ultimately promoting AF/ACM. We first discuss two pathways of mechano-transduction leading to nuclear signaling, altered transcription/translation, and remodeling. As reviewed by Saucerman et al. one path of mechano-transduction to the nucleus occurs via mechanical cues inducing this molecular signaling via sarcomeres, membrane channels, the extracellular matrix, the cytoskeleton, receptors, and integrins/dystrophin complex [18]. These mechanical cues employ nuclear signaling to regulate gene expression in physiological loading of the ventricular myocardium. With persistent and abnormal loading there is induction of maladaptive gene expression. Consequently, there is altered remodeling, hypertrophy, fibrosis, and alterations in the micro-environment [18], [166]. Based on observations of adverse structural remodeling and electrical instability observed in hypertrophic HCM and DCM, which are primarily linked to genetic variants expressing mutant sarcomere proteins (see Table 1), we focus here on sarcomere signaling. There is evidence that altered atrial sarcomere function promotes signaling pathways inducing fibrosis, inflammation, endothelial modifications, and release of prothrombotic factors and mechano-electrical signaling, ultimately leading to structural remodeling and electrophysiological instability [167], [168], [169], [170], [171], [172]. We and others have previously reviewed the role of sarcomeres in mechano-transduction via signals generated at the Z-disk, M-band, and titin involving release of transcription regulators that shuttle to nucleus; a direct connection to the nuclear envelope is via cytoskeletal elements, particularly desmin [14], [57], [173], [174]. It is also potentially relevant in AF/ACM that mutant cTnT linked to DCM and electrical abnormalities has been reported to enter the nucleus and regulate transcription epigenetically [175]. There is evidence of crosstalk between the myocytes and fibroblasts as well that is modified in AF [18], [176], [177].

A specific example of the broad effects of mechanical transduction arising from sarcomeres regulated by mutant proteins is our investigation of right atria of an HCM model (Tm-E180G) [169]. This model shows evidence of large RA thrombi, increased volume, and fibrosis [167], [169]. We employed 3D lightsheet microscopy and a workflow providing multiplex fluorescence detection of epitope markers of lymphatics and fibrotic tissue [169]. Our lightsheet measurements showed a significant modification in lymphatic vessel morphology correlated with the volume of fibrotic tissue. Inflammation and endothelial disorders are commonly found in familial HCM models, but the role of lymphatic modifications requires further study.

### 6.2 Hippo and Notch signaling in mechano-transduction/remodeling

The Hippo pathway and Notch signaling encompass other major mechanisms of mechano-transduction in cardiac myocytes. Notch and Hippo are understood to be significantly involved in cardiac development, silenced with maturation, but can be reactivated in mechanical stress on the adult myocardium [173], [178], [179], [180], [181], [182]. Reactivation of both Notch signaling and Hippo signaling has been implicated in HCM, DCM and AF [168], [173], [180], [182]. The Hippo pathway interrogates the micro-environment of multiple cell types including cardiac myocytes providing surveillance of the mechanical state. This surveillance engages signaling pathways critical to homeostasis of cardiac development, remodeling, and function and vulnerable to pathologies [168], [183]. The Hippo mechano-sensors regulate a stream of signaling affecting nuclear co-activators, YAP (yes associated protein) and its paralog TAZ, (transcriptional coactivator with PDZ-binding motif), modulating transcription. Adverse Hippo/YAP signaling has been reported in HCM, emphasizing a role for sarcomere signaling affecting this pathway [180], [184]. In an analysis of differentially expressed genes in human AF pathogenesis, Pan et al. also identified a role for Hippo Signaling as a pathway leading to promotion of TGF-β expression [181]. Pathways known to be dysregulated in HCM, include metabolic, inflammatory, and extracellular matrix pathways [173]. In the case DCM, we have reviewed a role for adverse Hippo signaling in its progression [185].

As reviewed in our perspective there is a strong potential for the role of Notch signaling and its intersection with Hippo signaling in arrhythmias, microvascular ischemia, and fibrosis [182]. Our studies discovered a potential link of Notch signaling to arrhythmias [183]. Promotion of Notch signaling related to HCM sarcomere mutations was identified in cardiac myocytes derived from human inducible pluripotent stem cell variants of TNNT2, which encodes cTnT, linked to arrhythmogenesis and sudden death [183]. We suggested that use of these derived cardiomyocytes may provide a predictor of susceptibility of sudden death. In the case of AF, it has been reported that atrial samples from patients with HF + AF, but not HF alone, that there is a specific activation of the Notch pathway in LA [178]. In a murine model transient activation of Notch induced a transcriptional profile like that of AF [178]. There is a need for further investigation of the role of adverse sarcomere function linked to Notch/Hippo signaling in predisposing both genetic and acquired AF/ACM.

## 7 A role for altered sarcomere function in adverse mechano-electrical coupling

Adverse mechano-electrical coupling caused by changes in sarcomere function, constitute a significant pathological pathway in AF/ACM [186]. Yet, maladaptive sarcomere function remains relatively underrepresented in current models of mechano-electrical signaling in AF/ACM. As an extension of Fig. 5, Fig. 6, we provide an overview of atrial mechano-electrical coupling mechanisms susceptible to disruption via modifications in sarcomere function and signaling. Key processes include influences of stretch and contractility modifications in channel function and capacitance, Ca^2+^ buffering, Ca^2+^ fluxes, and changes in energetics/reactive oxygen species. Notable mechanisms are alterations of sarcomere function that can significantly impact atrial filling, stretch, and elastance during the reservoir phase, as indicated by the dashed line in Fig. 2, Fig. 4.The mechanical state of diastolic sarcomeres plays a major role during this passive phase when intracellular Ca^2+^ concentrations are low. Impaired atrial diastole, resulting from sarcomeric protein mutations associated with HCM and DCM, proteolysis, oxidation, and PTMs, is a prevalent pathology observed in both genetic and acquired forms of AF/ACM [102], [187], [188]. At the bottom of Fig. 6 is a demonstration of this mechanism as determined by measurements, derived from the data reported by O'Neill et al. comparing LA strain in a normal individual and in a patient with ACM [119]. The strain during the reservoir and thus atrial deformation is most prominent during the reservoir phase. As a result of these sarcomere modifications affecting the reservoir phase, there is an altered atrial deformation during filling that affects stretch-dependent atrial channels resulting in after-depolarizations and generation of drifting electrical rotors [186]. Stretch dependent changes in membrane capacitance in the myocardium has also been reported to be associated with slowing of conduction with increased pre-load [189], [190] Modification of atrial myocytes by adverse, persistent changes in contractile state is also an important pathological mechanism affecting mechano-electrical signaling. As pointed out above, during local stretch of myocytes there is activation of stretch activated channels; sensitivity to the stretch increases in hypertrophied/failing myocytes [191]. It is apparent that this change in sensitivity could be due to multiple factors at the level of membrane but is also likely to be due to altered sarcomere contractility [191]. Effects of altered shear stress associated with altered contractility can also be important in AF/ACM. During each contraction and hemodynamic disturbance, cardiac myocytes are deformed by interlaminar fluid flow and myocardial layers sliding against each other [192], [193]. Interlaminar shear stress has been approximated in the adult rat atrial tissue indicating that changes in contractile state, pressure and volume may be an important cause of AF [194]. Changes in shear stress influence the recruitment of channels to the surface membrane from vesicles through a mechanism that relies on microtubules, integrins, focal adhesion kinase, and likely involves a network linked to the sarcomere contractile state. Moreover, mechano-signaling at the level of the sarcomeres, which is evident in HCM linked to sarcomere protein mutations, can affect pathways inducing fibrosis and inflammation, which have strong associations with AF/ACM [26], [195], [196].

Regulation of the kinetics of Ca^2+^ binding to cTnC can occur physiologically by multiple interactions among the myofilament proteins and thus engage normal mechano-electrical signaling. Buffering of cytoplasmic Ca^2+^, which is homeostatic in myocyte function and electrical stability, is vulnerable as a mechanism in AF [197]. Alterations at the level of thin filament proteins affecting cytoplasmic Ca^2+^ buffering demonstrate adverse mechano-electrical-signaling in AF/ACM. A specific example of disturbance in this regulation is evidence of altered Ca^2+^ buffering in human AF induced by proteolytic loss of myofilament proteins including cTnC with adverse effects on generation [29], [157]. In one study, atrial samples isolated from patients with persistent AF showed a degradation of cardiac TnC (cTnC) [157]. This loss of cTnC led to altered buffering of intracellular Ca^2+^, inducing spontaneous and arrhythmic Ca^2+^ release. These studies also reported a loss of cTnI with an increase in phosphorylation at the N-terminus [157]. These changes would also be expected to affect Ca^2+^-buffering by cTnC [198]. The lower passive tension in atrial titin vs. ventricular titin would also be expected to alter Ca-binding to TnC as discussed below [126]. There is also evidence that a transient mechanical stretch of cardiac sarcomeres induces Ca^2+^ dissociation from the myofilaments leading to induction of arrhythmias [199]. This release of Ca^2+^ from micro-domains due to contractility and effects at the mitochondrial/sarcoplasmic reticulum interface has been implicated in AF in an extensive review by Schonleitner et al. [200].

Oxidative stress has been also implicated in mechanisms associated with adverse sarcomere function. For example, generation of reactive oxygen species is well documented in HCM and affects the stiffness of titin via glutathionylation [42], [105], [201], [202]. The authors summarized mechano-energetic uncoupling with mismatches in energy demand and energy supply because of this viscous cycle of adverse signaling. Atrial economy of contraction (ATPase/tension) is 5-fold more efficient in ventricular myocytes compared to atrial myocytes [203]. Thus, mechano-energetic uncoupling may be more vulnerable in atrial tissue than ventricular tissue. An outcome of the depletion of antioxidative capacity is oxidative stress, known to modulate sarcomere regulation and dynamics, and likely to be a contributor to AF [105].

## 8 Sarcomere-focused treatment for atrial cardiomyopathies

Atrial fibrillation (AF) progression is strongly influenced by atrial cardiomyopathy (ACM), though direct clinical trials targeting ACM are limited. Preclinical and clinical evidence suggests sarcomere-targeting therapies—particularly myosin modulators—may modify atrial substrate and AF risk. Myosin activators such as danicamtiv and omecamtiv improve atrial and ventricular function, with mixed effects on AF outcomes depending on patient population. Myosin inhibitors (e.g., mavacamten, aficamten) show variable effects on atrial remodeling and AF incidence, with generally low AF risk in selected hypertrophic cardiomyopathy cohorts. In contrast, Ca^2+^ sensitizers have been limited by proarrhythmic effects. Overall, sarcomere-modifying therapies show promise but require further targeted clinical validation. A significant challenge is to tease out specificity for the sarcomeres in the atrial compartment.

AF recurrence rates remain high, and eventual progression to persistent/permanent AF is common. The contribution of the atrial substrate in propagating AF, and other atrial arrhythmias, and evidence for atrial cardiomyopathy (ACM) as a major driver of AF, has only recently been appreciated. Although there are no major clinical trials yet that directly test whether treating ACM can prevent AF onset/progression, there are several pre-clinical and clinical studies that suggest that sarcomere-targeting drugs especially modifiers of myosin activity, may play a role in modifying the atrial substrate and AF risk. Voors et al. showed that danicamtiv increased ATPase activity, increased Ca^2+^ sensitivity, increased left atrial (LA) emptying fraction, increased LA function index, and decreased LA minimal volume index [204]. This study was limited by the exclusion of patients with underlying atrial fibrillation; however, given the positive results on improving atrial performance, these data may serve as a rationale for a trial of danicamtiv in this population. Results of studies with omecamtiv mecarbil have given mixed results indicating the need for future clinical trials and a search for new sarcomere activators [205], [206]. Conversely, myosin inhibitors such as mavacamten and aficamten, both in clinical use, for the adults by reducing hypercontractility and improving cellular metabolism in HCM [207], [208]. New data from next-in-class cardiac myosin inhibitor aficamten appear to show a low incidence of atrial fibrillation in patients with obstructive HCM [208]. Although there is a rationale for their use in AF, thin filament Ca^2+^ sensitizers modifying thin filament function have not been successful in view of the induction of arrhythmias [51], [157], [209]. It is worth noting that, the presence of preexisting AF in these study patients suggests worse severity of disease overall. In summary, new sarcomere-modifying drugs hold promise for reversing atrial remodeling and a lower incidence of AF in selected populations. By individualizing sarcomere-modulating therapies to the underlying arrhythmogenic substrate, it is possible to simultaneously address ventricular and atrial cardiomyopathies. It will also be of interest to determine the effect of myosin activators on the sarcomere proteins and function of cardiac myocytes in the pulmonary vein sleeves [161]. Ectopic activity of the myocytes in these sleeves is causal in AF. Moreover, the regulation and isoform population of these sleeve sarcomeres differ from that in the atria.

## 9 Conclusions

We have reviewed literature indicating that proper regulation of sarcomere components—including regulatory unit composition, myosin head states, and mechano-biochemical signaling—is essential for atrial mechanical stability and maintenance of sinus rhythm. Interactions among titin, thick, and thin filaments alter diastolic behavior and Ca^2+^ sensitivity, creating conditions that promote atrial arrhythmogenesis. Modulating myosin activity or intracellular Ca^2+^ sensitivity may allow therapeutic “tuning” of the atrial sarcomere to reduce susceptibility to ACM and AF. Future clinical studies are needed to determine whether targeting these sarcomere mechanisms can prevent or reverse atrial cardiomyopathy.

## CRediT authorship contribution statement

**Mark D. McCauley:** Writing – review & editing, Writing – original draft, Methodology, Investigation, Conceptualization. **R. John Solaro:** Writing – review & editing, Writing – original draft, Project administration, Methodology, Investigation, Conceptualization.

## Declaration of competing interest

The authors declare that they have no conflicts of interest to report regarding the present manuscript.
